# Perceptual learning improves discrimination but does not reduce distortions in appearance

**DOI:** 10.1371/journal.pcbi.1012980

**Published:** 2025-04-15

**Authors:** Sarit F.A. Szpiro, Charlie S. Burlingham, Eero P. Simoncelli, Marisa Carrasco

**Affiliations:** 1 Department of Special Education, Faculty of Education, University of Haifa, The Edmond J. Safra Brain Research Center, University of Haifa, Haifa, Israel; 2 Department of Psychology, New York University, New York, New York, United States of America; 3 Center for Neural Science, New York University, New York, New York, United States of America; 4 Courant Institute of Mathematical Sciences, New York University, New York, New York, United States of America; 5 Flatiron Institute, Simons Foundation, New York, New York, United States of America; UC Irvine: University of California Irvine, UNITED STATES OF AMERICA

## Abstract

Human perceptual sensitivity often improves with training, a phenomenon known as “perceptual learning.” Another important perceptual dimension is appearance, the subjective sense of stimulus magnitude. Are training-induced improvements in sensitivity accompanied by more accurate appearance? Here, we examined this question by measuring both discrimination (sensitivity) and estimation (appearance) responses to near-horizontal motion directions, which are known to be repulsed away from horizontal. Participants performed discrimination and estimation tasks before and after training in either the discrimination or the estimation task or none (control group). Human observers who trained in either discrimination or estimation exhibited improvements in discrimination accuracy, but estimation repulsion did not decrease; instead, it either persisted or increased. Hence, distortions in perception can be exacerbated after perceptual learning. We developed a computational observer model in which perceptual learning arises from increases in the precision of underlying neural representations, which explains this counterintuitive finding. For each observer, the fitted model accounted for discrimination performance, the distribution of estimates, and their changes with training. Our empirical findings and modeling suggest that learning enhances distinctions between categories, a potentially important aspect of real-world perception and perceptual learning.

## Author summary

Practicing a perceptual task often enhances the detection and discrimination of relevant visual information, a phenomenon known as perceptual learning, which reflects neural plasticity in the adult brain. For instance, a trained professional can easily identify critical details in fetal ultrasound images, whereas novice parents may perceive little meaning in the same images. Another crucial aspect of perception is appearance—the subjective visual experience—which does not always match the physical stimulus. It remains unknown whether and how appearance changes after perceptual learning. For instance, if the appearance of ultrasound images differs for trained and untrained individuals. In this study, we investigated how training modifies both performance and the appearance of motion stimuli that are initially perceived as distorted. While training improved sensitivity, it did not reduce the perceived distortions in appearance and, in many cases even increased them. Our findings reveal a distinction between performance and appearance in perceptual learning and suggest interactions between sensory and cognitive processes. To explain these results, we developed a computational model that links learning, categorization, and efficient encoding. The model suggests that improvements in precision can account for the observed coupling between enhanced sensitivity and distortions in appearance following training.

## Introduction

One of the most remarkable forms of adult brain plasticity is the capacity to develop perceptual expertise. Perceptual learning (PL) is defined as long-lasting improvements in the performance of perceptual tasks following practice (for reviews, see [1–3]). PL has been documented in every sensory modality (see review, [4]), and has been studied using detection and discrimination tasks in behavior [5–11], electrophysiology [12–18], neuroimaging [19–23], and computational modeling [24–28].

Most studies of perceptual learning have focused on task performance (e.g., improved accuracy in judging a given stimulus attribute or reduced thresholds in detection and discrimination tasks). However, another critical aspect of perception is *appearance*—the subjectively perceived magnitude of a stimulus attribute, which can be assessed via estimation tasks. Stimulus appearance is often systematically distorted relative to physical stimulus properties. For example, although discrimination accuracy is better near cardinal (horizontal or vertical) than near oblique orientations (known as the “oblique effect”), orientations near horizontal appear repulsed away from horizontal, deviating from their physical value [29–32]. But the effects of PL on appearance, and the relation of these effects to those on sensitivity, remain largely unknown. The conjoint effect of PL on performance and appearance has implications for theories and computational models [5,33,34,67], neurophysiology [35], and philosophy [36] of perception and learning. Moreover, this conjoint effect can inform clinical rehabilitation protocols, such as those used for people with amblyopia [37–39] and with cortical blindness [40–43], and for developing effective training protocols for acquiring perceptual expertise (e.g., for radiologists [44]).

One might suspect that PL could reduce estimation biases. However, the only study to date that has investigated motion appearance and PL found that training (without feedback) to estimate nearly horizontal motion *increased* repulsive biases away from horizontal, as measured through either explicit estimates of motion direction or the direction of smooth pursuit eye movements [7]. This study, however, did not examine the critical question of how training on a discrimination task—used in nearly all PL studies and rehabilitation protocols—affects estimation biases. Assessing this would broaden our understanding of how PL affects perceptual representations.

**Fig 1:**
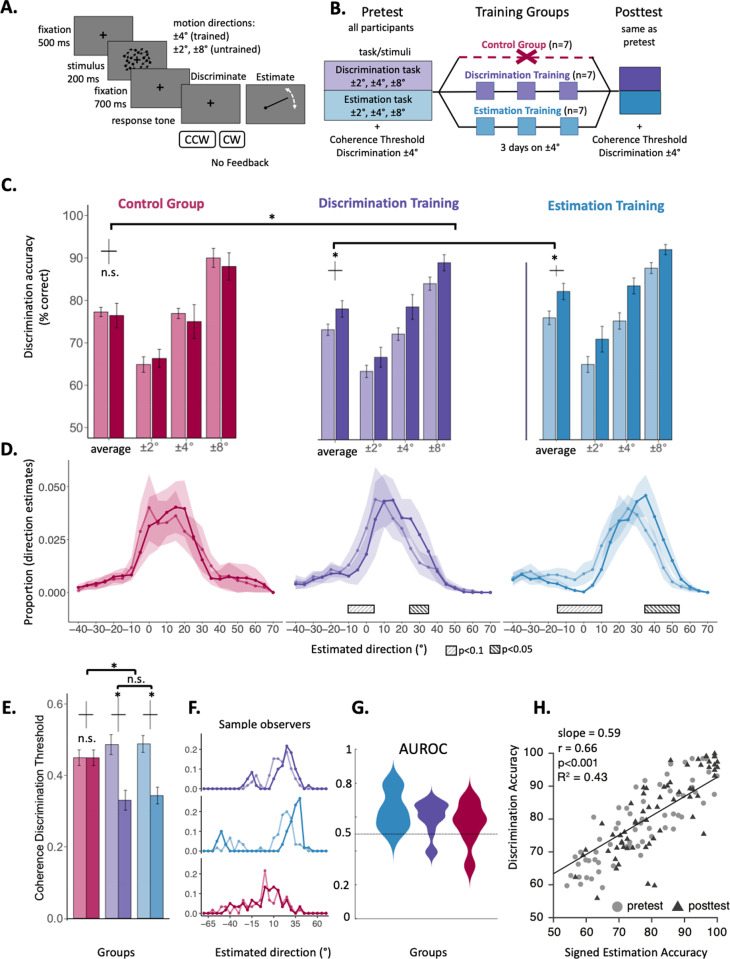
Experimental protocol and behavioral evidence of perceptual learning. (A) Trial sequence. (B) Training procedure for three groups (Control, Discrimination training, and Estimation training). (C) Average discrimination accuracy across groups (indicated with distinct colors, one per training group) at PRE- and post-test (unsaturated vs. saturated colors, respectively) across stimulus directions. Errors bars represent ±1 standard error of the mean with Morey’s correction. (D) Illustration of estimate distributions at the group level. The mean estimate proportion for each group at pre-test versus post-test is shown (unsaturated vs. saturated lines, respectively), collapsed across directions and averaged over 5° bins. The shaded area represents the standard error of the mean. Significant clusters for the main effect of PRE/post training, as determined by cluster mass permutation two-tailed tests, are indicated with rectangles (p < 0.05 and p < 0.1). Note that these estimate plots depict the group-level estimate distributions for illustration purposes; however, the analysis was based on a mixed-effects linear model that accounts for individual variability across groups and participants, thereby addressing the often bi-modal estimate distributions observed in individual participants (Fig A in S1 Text, Fig 2). (E) Coherence discrimination threshold for each training group at pre-test versus post-test (unsaturated vs. saturated lines, respectively; crosses indicate the error bars between pretest and posttest within each group). For the discrimination and estimation groups the coherence discrimination thresholds decreased from the pre-test to the post-test sessions p < .001), whereas for the control group that was not the case (p = .99). There was an interaction between control and training groups (p = 0.009), but not between the two training groups (F < 1). (F) Sample observers’ estimate distributions for each group at pre-test versus post-test (unsaturated vs. saturated lines, respectively). (G) Violin plots for each group showing AUROC values. The horizontal line at 0.5 indicates no separation between PRE- and post-test distributions. Values above 0.5 indicate a repulsion of post-test estimates from pre-test estimates, away from horizontal. (H) Correlation between discrimination accuracy and signed estimation accuracy (percentage of direction estimates consistent with the correct up/down category) across all observers at pre-test (light circles) and post-test (dark triangles).

Here, we investigated the behavioral consequences of training with either discrimination or estimation on both tasks. Observers viewed near-horizontal motion directions and were initially tested (“pre-training”) on both a discrimination task (indicate “clockwise” or “counterclockwise” direction) and an estimation task (indicate perceived motion direction by adjusting the orientation of a line; Fig 1A). Because we were interested in unsupervised learning, no feedback was given for either the discrimination or estimation tasks, as feedback is not necessary for PL to occur [45–49] and could affect the subjective reports rather than appearance (see Discussion).

All observers exhibited substantial repulsive biases in the estimation task (e.g., for a 4º motion stimulus they made a 12º estimate), consistent with previous studies of reference repulsion [29,30]. Similar to prior research [33,50–52], we found that observers also made estimates that were on the other side of the horizontal boundary (e.g., for a + 4º stimulus, they made a -12º “misclassified” estimate), resulting in a bimodal distribution of estimates. After three days of training on either task, as expected, participants in both groups showed a comparable improvement in discrimination accuracy, and a reduction in motion noise coherence thresholds. Notably, while training decreased the frequency of misclassified estimates, it did not reduce the repulsive bias and, for some participants, even increased it. Thus, PL enhanced performance but failed to diminish – and, in some cases, even exacerbated – distortions in stimulus appearance.

To explain these results, we developed a computational observer model that predicts discrimination accuracy and the distribution of estimation responses before and after trai ning. The model relies on three primary assumptions: (1) the internal representation favors cardinal motion directions, which are prevalent in the natural environment; (2) in the estimation task, observers implicitly categorize motion and condition their estimates on this categorization; and (3) both types of training increase the precision of representation for trained motions. We find that the model simulations, fitted to individual observer data, can account for discrimination accuracy and estimate distributions (including biases and bimodality), both before and after training. Our model establishes a link between enhanced sensitivity and perceptual biases. Thus, PL leads to a more precise representation that supports improved discrimination performance while contributing to the distorted appearance of stimulus magnitude.

## Results

To investigate how PL modifies both discrimination ability and appearance, we asked observers to judge random dot stimuli moving in near-horizontal directions. Observers responded in one of two tasks: a discrimination task, where they indicated whether the motion was “clockwise” or “counterclockwise” relative to rightward horizontal, or an estimation task, where they adjusted the orientation of a line to match the perceived direction of motion. Initially, all observers performed both tasks for motion directions of ± 2°, ± 4°, and ± 8° relative to horizontal (Fig 1A, B). Following this pre-test, one group (n = 7) was trained on the ± 4° estimation task, while another group (n = 7) was trained on the ± 4° discrimination task, for three consecutive days. A third group, the control group (n = 7), performed only the pre-training tests and did not receive any additional training over the next three days. Finally, in the post-test, all three groups of observers were tested on both tasks. No feedback was provided in any condition, either during training or testing.

All stimuli consisted of a mix of dots moving coherently in a common direction and a subset moving randomly. Before the experiment, each observer’s noise coherence threshold (the proportion of coherent dots needed to achieve 75% discrimination accuracy) was obtained for the ± 4° motion directions using a staircase procedure. This threshold value was subsequently used in testing and training sessions, and the coherence threshold was re-assessed after the post-test was completed. In the pre-test, neither discrimination accuracy nor estimation judgments interacted with training group for either stimulus motion direction (both F(4,36)<1), indicating that the groups had comparable performance in both tasks prior to training.

### Discrimination results

#### Perceptual learning improved discrimination accuracy.

For both training groups, PL improved discrimination of motion direction (Fig 1C), consistent with prior studies [6,53]. A mixed-design ANOVA revealed a 2-way interaction (Training groups vs. Control group X Session: F(1,19)=5.79, p = 0.02): Discrimination accuracy improved between the pre-training and the post-training sessions for observers who trained (Session: F(1,12)=17.35, p = .001), and improved similarly across both training tasks (Training Task x Session: F (1,12)<1) and motion directions (Training Task X Session X Direction: F (2,24)<1). However, accuracy remained unchanged for observers in the group who did not train (Control group: F(1,6)<1; see individual subject data in Fig C in S1 Text). In summary, discrimination improved regardless of the training task and to a similar extent for both trained and untrained motion directions.

#### Perceptual learning decreased noise thresholds.

We analyzed noise coherence thresholds before and after training to determine whether there was an improvement in the signal-to-noise ratio, as has been found in previous PL research (e.g., [54–58]). Due to a technical issue, noise thresholds at post-test were not available for three observers, so we focus our analysis on the remaining 18 participants. There was a 2-way interaction between group and session (Training groups vs. Control group X Session: F(1,16)=8.9, p = 0.009; Fig 1E). In the control group, without training, coherence thresholds did not significantly change between pre-test and post-test sessions, from 47.3% to 44.9%, (t(4)=0.04, p = 0.99). In contrast, training reduced coherence thresholds to a similar degree for both training groups (*Session*: F(1,10)=22.64, p < 0.001; *Training Task X Session*: F(1,10)<1): For estimation training from 48.8% to 34.3% (t(6)=3.55, p = 0.01); for discrimination training, from 47.1% to 33.1% (t(5)=2.97, p = 0.03, Fig 1E; see individual subject data in Fig C in S1 Text). Across observers, decrements in noise coherence thresholds between post-test and pre-test were correlated with increments in discrimination accuracy between post-test and pre-test (r = -0.66, p < 0.001).

### Estimation results

#### Bimodality in the estimate distribution.

**Fig 2:**
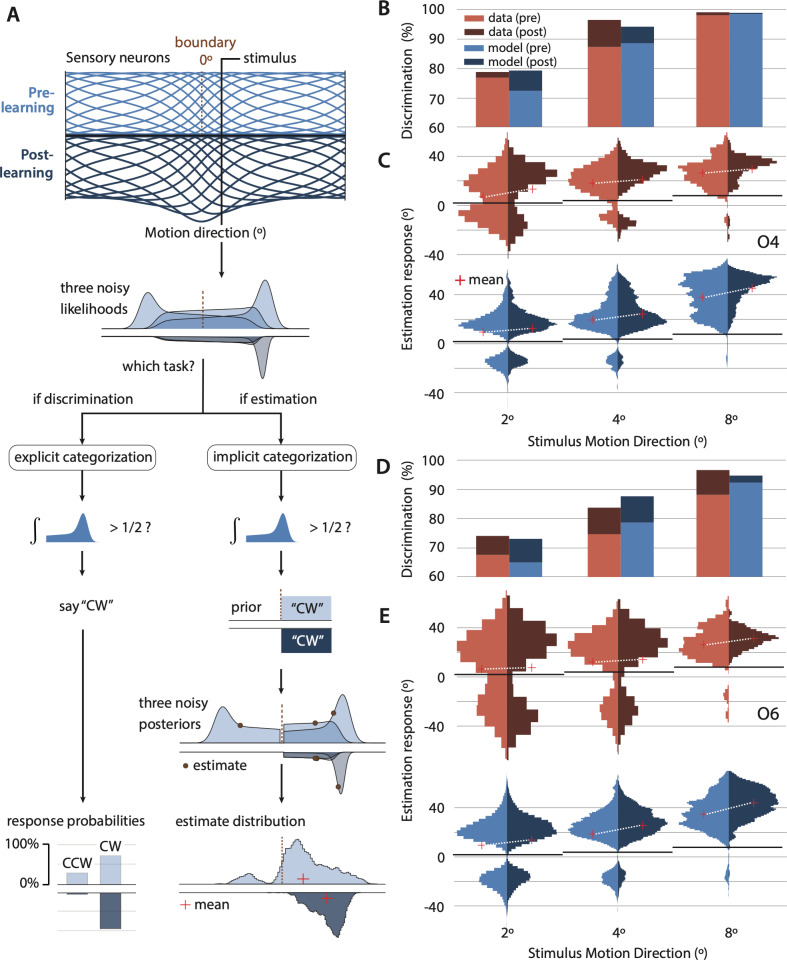
Observer model and human/model behavior for two observers. (A) A population of tuned sensory neurons encodes motion direction (tuning curves representing the mean stimulus response; Before training - light blue, upper panels; After training – dark blue, inverted, lower panels). The population is warped such that more neurons represent near-horizontal motion directions, even before training consistent with efficient encoding [65,67]. Training increases the gain of the neurons encoding the trained stimuli (±4°), rescaling their tuning curves (inverted, dark blue). For each trial, spike counts for each neuron are drawn from independent Poisson distributions, with the firing rate determined by the value of the tuning curve at the stimulus’s motion direction. The decoder computes the likelihood of different motion directions based on these noisy responses and is assumed to be “unaware” of the warping in motion space. As a result, the likelihood functions are skewed (3 examples shown). Due to sensory noise, likelihoods fluctuate across trials, with modes occasionally falling on the opposite side of the boundary relative to the stimulus. Regardless of the task, the observer performs a discrimination judgment by comparing the mass of the likelihoods on the two sides of the boundary. For the discrimination task, this answer is reported. For the estimation task, this “implicit” discrimination judgment conditions the upcoming estimate. Specifically, estimates (3 examples indicated with black points) are computed as the mean of the portion of the likelihood on the side of the boundary corresponding to the chosen category. The mean estimate (red cross) is biased away from the true stimulus by both efficient encoding (warping) and the conditional judgment. When, by chance, the likelihood falls on the incorrect side of the boundary, its corresponding estimate contributes to a second mode in the estimate distribution, which we label as “misclassified.” The training-induced increase in sensory gain generates fewer likelihoods on the wrong side of the boundary and interacts with the efficient coding and unaware decoding to reduce internal evidence for motion near the horizontal boundary (i.e., “boundary avoidance”). This decreases misclassifications of motion directions (i.e., increases discrimination accuracy) and amplifies estimation bias. (B,D) Comparison of human and model behavior in the discrimination task for two representative observers in the estimation training group (O4, O6). Bar height represents discrimination accuracy (% correct) across trials (N = 120). (C,E) Comparison of human and model behavior in the estimation task. Histograms of estimates (N = 60). The red cross indicates the mean of the estimate distribution. The white line indicates the change in the mean with learning. The black line represents the stimulus motion direction. Note the bimodal shape of the estimate distribution (arising from the implicit discrimination judgement) and its dependence on learning and stimulus motion direction.

Analyzing responses in the estimation task, we found that the distributions of estimates often exhibited bimodality. Typically, there was a primary mode on the ‘correct’ side of the boundary (i.e., corresponding to the true category of the stimulus, e.g., “CW”), though this mode was centered around a biased value (e.g., 15° for observer O4 and directions of =+4°and -4° collapsed such that estimates with a positive sign correspond to the correct category; Fig 2). Additionally, a secondary mode was observed on the ‘incorrect’ side of the boundary (e.g., “CCW”), centered approximately at the negative of the primary mode (e.g., -9° for observer O4 estimates with a negative sign indicate the incorrect category; Fig 2). In a few cases, there was also a third mode centered near the horizontal boundary (Fig A in S1 Text). We quantified the shape of these distributions by fitting them with Gaussian Mixture Models containing between one and three components. In the pre-test, over half of the estimate distributions were best classified as bimodal (63%, across all observers and directions), and the remainder were classified as either unimodal (24%) or tri-modal (13%) (Fig A in S1 Text, and see qq-plots of distributions in Fig B in S1 Text).

Bimodal estimate distributions, as found in the current study, have been observed in experiments that require an explicit categorization judgment prior to the estimation response [50–52]. Based on the similarities between these past datasets and ours, we hypothesized that observers in our study performed an *implicit* categorization (‘CW’ versus ‘CCW’), which influenced their subsequent estimation judgment, even though they were not explicitly instructed to do so [33,50]. To test this hypothesis, we implemented this assumption in an observer model and showed that it is essential for the model to reproduce the shape of the estimate distributions and their changes with PL.

#### Perceptual learning modified the estimate distributions from pre-test to post-test.

Our results revealed that estimate distributions were often bimodal (on each side of the horizontal discrimination boundary) consistent with previous studies [33,50–52], and changed in shape substantially between pre-test and post-test and across stimulus directions. These shape changes included changes in the tails (e.g., slowness), lateral shifts in each sub-distribution, and shifts in the relative mass of the two sub-distributions. Due to these changes and the bimodality of the estimate distributions, classic measures of central tendency (e.g., mean, median, mode) and those derived from them (e.g., bias), do not provide effective measures of PL and transfer effects. Thus, to measure the influence of PL on motion estimation, we implemented three complementary non-parametric methods to characterize the specific changes observed between pre-test and post-test: (1) a cluster-mass permutation test, to quantify learning-induced changes in the estimate distribution, including possible changes in slowness and tail thickness; (2) signed estimation accuracy –the proportion of estimates on the correct side of the discrimination boundary – to quantify the density increment of correctly classified estimation judgments after training; (3) an area-under-the-ROC-curve (AUROC) analysis on the correctly classified estimates only, to measure lateral shifts and tail changes in that sub-distribution. Following these analyses, we demonstrated that our observer model captures the various effects in our data.

***Cluster Mass Test***. We examined the difference in the distributions of estimation responses between the pre-test and post-test. Given that distributions were in most cases multi modal, we employed permutation-based cluster mass tests [59] to identify clusters of adjacent bins of motion directions with a significant difference in estimate counts between pre-test and post-test (see Cluster-mass permutation tests in Appendix A in S1 Text). This approach does not assume any specific shape of the estimate distribution, which varied among participants, across motion directions, and between the PRE- vs. post-test (see Fig A in S1 Text and results section “The model captures individual differences among observers”). Briefly, we binned estimate response counts into 5° motion direction bins for each participant in the pre-test and post-test. We then used a Poisson generalized linear mixed model (GLMM) to identify bins where the counts of estimate responses were significantly different between pre-test and post-test by permuting the PRE/post labels. The GLMM included a random intercept term for each participant to account for individual differences in the distributions. We then identified clusters of adjacent bins showing consistent changes compared to a randomly permuted distribution of clusters. This analysis enabled us to identify clusters of bins with significant changes in the counts of estimate responses between pre-test and post-test for each training group, as well as interactions between training groups and the control.

We found that there was a significant interaction (p < .05) between the training groups and the control group (by permuting group assignments and PRE-/post-test session results; see Methods). In the training groups – but not in the control group – significant clusters emerged in the post-test where the estimate distributions differed from the pre-test estimate distribution. For the discrimination training group, the significant cluster was at 25°–35°, and for estimation training group, the cluster was at 35°–55°; Fig 1D). Importantly, these significant clusters indicate that, when examining the distribution of estimate responses, the counts of estimates in these bins increased significantly from pre-test to post-test. In addition, marginally significant clusters also emerged on the *incorrect side* of the category (negative directions in Fig 1D), where the count of estimates decreased at post-test after training. For the discrimination training group, the cluster was at –10° to –5°, and for the estimation training group, the cluster was at –15° to –10° (Fig 1D). In sum, these clusters suggest that PL affects estimation by either (a) shifting a proportion of estimates from the incorrect side of the boundary to the correct side; (b) increasing the repulsion of estimates on the correct side of the boundary (e.g., an estimate response of 9° at pre-test versus 15° at post-test); or (c) some combination of the two. The following analyses directly examine these possibilities.

***Signed Estimation Accuracy***. We examined the proportion of ‘correct’ estimates–those that fall within the true stimulus category (e.g., “CW” or “CCW”), which we termed “signed estimation accuracy”. For example, for a presented direction of 4°, an estimate response of 6° is on the correct side of the boundary, whereas an estimate of -5° is on the incorrect side. Quantifying whether an estimate response fell in the correct or incorrect category yielded the percent of estimates that matched the true stimulus category (up/down). This approach allows us to assess whether the proportion of estimate responses in the correct category changed after training. Training significantly increased signed estimation accuracy in both training groups, regardless of the training task and across directions (*Session*: F(1,12)=25.9, p < 0.001; *Session X Direction X Training Task*: F(2,24)<1). There was a marginally significant interaction on signed estimation accuracy (*Training groups vs. Control group X Session*: F(2,38)=2.85 p < 0.1). In the control group, signed estimation accuracy did not change significantly (*Session*: F(1,6)= 3.45, p = 0.112; *Session X Direction*: F(2,12)<1; see individual subject data in Fig C in S1 Text). Thus, training increased the proportion of estimate responses on the correct side of the boundary, effectively reducing the probability of misclassifying the stimulus direction into the incorrect category in the estimation task.

We also examined whether the proportion of ‘correct’ estimates, or signed estimation accuracy, is related to discrimination accuracy. We found a strong correlation between signed estimation accuracy and discrimination accuracy, collapsed across motion direction and training group for both testing sessions (overall: r = 0.66, p < 1x10^-6^; pre-test: r = 0.84; p < 1x10^-6^; post-test: r = 0.82, p < 1x10^-6^, Fig 1H), with no significant difference between sessions (Williams test: p = 0.766). These strong correlations are consistent with the hypothesis that observers implicitly categorized the stimulus in the estimation task.

***AUROC***. To test whether training shifted the correctly classified estimates further away from horizontal and from pre-test responses (specifically away from horizontal), we conducted an area under the receiver operating characteristic curve (AUROC) analysis [60] on the estimates on the correct side of the boundary. AUROC non-parametrically quantifies the separation between the pre-test and post-test distributions by comparing the cumulative probabilities of each distribution (see Methods for details). Here, the AUROC value reflects the shift between pre-test and post in the sub-distribution of correctly classified estimates, regardless of the number of correct -estimates in each. Therefore, AUROC is invariant to any potential increases in the number of correct estimates (i.e., it is unaffected by changes in signed estimation accuracy; see above). At pre-test, estimate distributions were repelled from horizontal, as expected from the reference repulsion effect [29,30]. Therefore, a shift could indicate movement either away from pre-test estimates and horizontal (i.e., increased repulsion) or toward pre-test estimates and horizontal (i.e., decreased repulsions). Each AUROC value quantifies the separation between the distributions: a value of 0.5 indicates complete overlap between pre-test and post-test distributions; values above 0.5 reflect a shift *away* from pre-test and from horizontal; and values below 0.5 reflect a shift toward pre-test and toward horizontal.

For each stimulus direction and observer, we computed the AUROC between the pre-test and post-test distributions (Fig 1G). Overall, there was a significant correlation between individuals’ posttest– pretest discrimination accuracy with the corresponding AUROC values (r = 0.27, p = 0.03). We tested whether the AUROC values significantly differed from 0.5 (i.e., no shift or complete overlap). For observers who trained, there was a significant shift between PRE- and post-test distributions: the AUROC was significantly above 0.5 for the training task overall (t(41)=7.09, p < 0.001) and for each training group individually (Discrimination Training: t(20)=4.35, p < 0.001; Estimation training: t(20)=5.71, p < 0.001). For the control observers, the estimate distributions also shifted, albeit to a lesser degree (t(20)=2.14, p = 0.04) and the magnitude of the shift was marginally smaller than that for the trained observers (Training groups vs. Control group t(61)=1.92, p = 0.059). The directions of these significant shifts were all away from horizontal and from the true stimuli. Examining the AUROC for each participant who trained on either discrimination or estimation, we observed that estimation responses did not become more veridical for 13 out of 14 observers. Surprisingly, estimation repulsions significantly increased in 7 of these 14 observers (Fig D in S1 Text). In the control group, 2 out of 7 observers showed an increase in AUROC values. In sum, estimates were not closer to the stimulus values after training; in some cases, they were significantly repulsed further from pre-test and from the classification boundary. Together, these results demonstrate that, signed estimation accuracy increased after training, and estimates that were repulsed at pre-test were not closer to the true stimulus values.

### Observer model

How does PL simultaneously improve discrimination accuracy and modify the estimate distribution? To explore these intriguing findings, we developed an observer model that performs and learns both tasks. The model incorporates three key mechanisms essential for explaining behavior: (1) Neural tuning preferences for cardinal motion, i.e., efficient encoding of motion directions, where more resources are allocated to directions that are more common in the natural environment; (2) Conditional inference based on implicit categorization – observers implicitly categorize motion and then condition their estimates based on their categorical decision; (3) Gain modulation – increased precision in the internal representation of trained motions in both tasks.

Only gain modulation was assumed to change with training. We analyzed the model’s ability to predict pre-training behavior and PL effects, specifically the differences between post- and pre-training behavior. In practice, we used the model to simulate either an estimation or discrimination response on each trial, based on the stimulus’s motion direction. We fit the model to the *entire* distribution of estimates as well as discrimination accuracy, both PRE- and post- training, minimizing the differences between the data and the model. We found that neural preferences for cardinal motion and implicit categorization allowed the model to explain behaviors present even before training (such as estimate bimodality and large overestimation of motion direction), and that gain modulation was crucial for explaining the observed PL effects, including increases in discrimination accuracy and changes in the estimate distribution.

Specifically, the observer model performs inference based on a probabilistic neural population code [51,61,62]. In the encoding stage, a stimulus elicits spikes in a population of motion-direction-sensitive neurons, with independent and identically distributed (i.i.d.). Poisson noise, resulting in a noisy population response on each trial. The neurons’ tuning curves tile the space of motion directions, but are warped to over-represent directions near horizontal (Fig 2A; [63–65]), as observed in physiological recordings [66]. This inhomogeneity is consistent with theories of coding efficiency, where more neural resources are devoted to cardinal directions due to their greater prevalence in the environment [67]. We assumed that the decoding stage is unaware of this warping [68] and, as a result, “assumes” that the encoding population is equi-spaced, leading to perceptual biases away from the horizontal boundary (i.e., “boundary avoidance”). Given that behavioral performance was similar across the two training groups, we hypothesized that PL arose from an increase in the precision of the internal representation of motion direction, irrespective of the training group. We modeled this assumption by applying a gain increase to the sensory neurons encoding the training stimuli (±4º).

The observer model makes either a discrimination or estimation judgment, depending on the task. For discrimination, the model reports “counterclockwise” if the majority of the internal evidence (likelihood function) lies counterclockwise of the discrimination boundary, and “clockwise” if it lies on the other side. For estimation, the model performs an implicit categorization (using the same discrimination rule), discards internal evidence inconsistent with the chosen category, and reports the mean of the remaining evidence as its estimate ([33]; see Methods for implementation details). We also assume that the behavioral response itself is noisy in both tasks: additive Gaussian motor noise in the estimation task, and lapse rate in the discrimination task.

#### Model behavior reproduces increased discrimination accuracy and shifts in distributions after PL.

For each observer, we fit the model to all behavioral data simultaneously: PRE- and post-learning discrimination accuracies, along with the full estimate distributions for all stimuli (±2, ± 4, and ± 8º). This process provided parameter estimates shared between the two tasks, as well as model behavior for each task separately (see Methods: Parameter Estimation).

**Fig 3:**
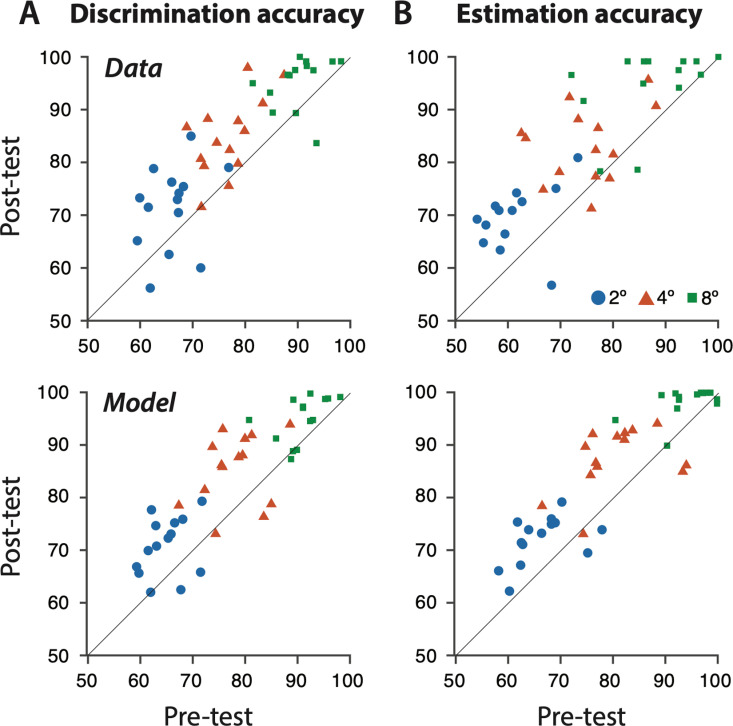
Comparison of human and model performance, over observers trained on both tasks. (A) PRE- vs. post-test discrimination accuracy. Top row, human behavioral data. Bottom row, model behavior. Each symbol represents the average discrimination accuracy for one observer, for the 2°, 4°, or 8° stimuli (blue circles, red triangles, green squares, respectively). (B) PRE- vs. post-test signed estimation accuracy (% of estimates falling on same side of the discrimination boundary as the stimulus motion direction). Note the similarity between discrimination and signed estimation accuracy in both the data and the model (see also Fig E-A).

The model behavior reproduced many aspects of observer behavior, including training-induced increases in discrimination accuracy (Fig 3A) and signed estimation accuracy (Fig 3B), as well as a strong correlation between discrimination accuracy and signed estimation accuracy before and after learning (Fig 1H; Figs E-B in S1 Text), and overestimation (Figs E-C in S1 Text). Furthermore, the model quantitatively reproduced the transfer of performance from the trained stimuli (±4º) to the other tested stimuli (±2 and ± 8º; Figs 2C and 3), including the scaling of discrimination accuracy (Fig 3A) and increases in signed estimation accuracy (Fig 2C). Specifically, after learning, the model produced fewer estimates inconsistent with the true category of the stimulus, which matched the human observers’ estimate distributions. Across observers, the model explained 85% of the variance in discrimination accuracy (p < 1x10^-6^), 52% of the variance in signed estimation accuracy (p < 1x10^-6^; Fig 3), and 44% of the variance in mean estimation (p < 1x10^-6^; Figs E-C in S1 Text).

#### The model captures individual differences among observers.

The model accounted for large individual differences in behavioral performance, many of which were present even before training. These differences included nearly unbiased estimation judgments for some observers (e.g., Figs E-A, O12, O15 in S1 Text) and highly biased estimation judgments for others (e.g., Fig 2: O4, O6; Figs E-A: O7 in S1 Text). These differences corresponded to varying numbers of neurons preferring cardinal motion (Fig L in S1 Text). Additionally, observers showed variability in discrimination accuracy, with some exhibiting low discrimination accuracy (e.g., Fig E-A: O7, O12 in S1 Text) and others high discrimination accuracy (e.g., Fig 2: O4; Figs E-A: O13 in S1 Text), corresponding to higher or lower levels of sensory noise, respectively. For observers with minimal warping or boundary avoidance, corresponding to a flatter environmental prior for motion directions (Fig E-A, O12, O15 in S1 Text), the model behavior resembled that of existing models of conditional inference [33,50].

The model also accounted for individual differences in the effects of PL on behavior, including notable increases in signed estimation accuracy (Fig 2: O4, O6), shifts in the sub-distribution (Figs E-A: O13 vs. O7 in S1 Text), and varying increases in discrimination accuracy (Figs E-A, O13 vs. O15 in S1 Text), ranging from small to large. These changes corresponded mechanistically to decreases in sensory noise with learning, which interacted with individual differences in sensory noise and neural preferences for cardinal vs. oblique motion that each observer had on day 1 of the experiment (see above).

The model also reproduced the variability in estimates observed in the control group (Fig F in S1 Text), as well as the clear signs of implicit categorization, including large overestimation both PRE- and post-training, and a tight correlation between estimation accuracy and discrimination accuracy.

#### Importance of model components in explaining human behavior.

We used model comparisons to qualitatively assess the causal contributions of each model component to the overall behavior (see Appendix B Reduced Models in S1 Text). In our model, PL arises from an increase in the precision of the representation of the training stimuli, which we implemented by increasing the gain of the sensory neurons encoding the training stimuli. When we removed the gain modulation (Fig G in S1 Text), the model’s behavior became comparable that of the observers in the control group (Fig F in S1 Text). Note, however, that other mechanisms can produce similar effects. In particular, a model variant in which gain modulation was replaced with tuning changes (such that more neurons in the population encode the trained directions) also captured many features of the data (see Appendix B in S1 Text; Fig H in S1 Text). We focused our analysis on the gain change model because it provided the best fit to the data, was simpler, and has stronger support from prior empirical literature [16,23,69].

When we removed the efficient encoding component (implemented through warping of the tuning curves), PRE- and post-training estimation biases showed the right pattern across stimuli and PRE- vs. post learning (Fig I in S1 Text) but were much smaller than those observed in the human data. On its own, the efficient encoding step does produce biased estimates (due to the asymmetric posterior), as found in previous studies [34,67,70]. Yet, the magnitude of this bias is limited by the width of the likelihood function (which, in our model, arises from a combination of gain, number of neurons, and tuning curve width; [65])). We concluded that efficient encoding enabled the model to explain both the magnitude of overestimation and the misclassified estimates observed far from the boundary.

When we removed the conditional inference (implicit categorization) step, the estimate distributions became unimodal, in contrast to the bimodal distributions observed in most of the data (Fig J in S1 Text). In this model variant, much of the likelihood function fell on the side of the discrimination boundary inconsistent with the true stimulus category, contributing to the left tail (bottom of Fig J in S1 Text) of a now unimodal, higher variance estimate distribution (Fig J in S1 Text) — instead of contributing to a separate sub-distribution, as occurs when conditional inference is used (Fig 2E). Thus, conditional inference allowed the model to explain the distinct bimodality of the estimate distribution.

Quantitative model comparisons confirmed our observations about each model variant (see in S1 Text). We compared the models using two metrics: one was the goodness-of-fit of each model (i.e., in-sample loss), and the other was generalization performance (i.e., out-of-sample cross-validated loss). Each model variant, including the reduced models and the tuning changes models, provided a worse fit to the data than the full model (Figs I-A in S1 Text). The generalization performance of the full model, the no-conditional-inference model, and the tuning-change model (“TC”) were similarly good, with the other models being much worse (Figs I-B in S1 Text). However, only the full model and tuning-change model (“TC”, which replaced gain changes in the full model with tuning changes) could reproduce the bimodal estimation data characteristic of human observers, suggesting that the no-conditional-inference model is inadequate.

## Discussion

The signature characteristic of PL is training-induced performance improvement, most commonly associated with a discrimination task [1,6–9,11,45,53–56]. We asked how training would concurrently affect stimulus appearance. Whereas one might have expected that improvement in discrimination accuracy would be accompanied by reduced estimation biases (i.e., learning would lead to a more veridical representation), our data did not support this hypothesis. Although participants who trained on either a discrimination or estimation task showed improvement in discrimination accuracy and coherence thresholds, but they also exhibited substantial repulsive biases that were present prior to training (Fig 1D and 1F), which did not diminish after training, they either remained or increased. Additionally, although training was restricted only to the + 4° and -4° directions, these effects transferred to untrained nearby directions (+2° and + 8°, and -2° and -8°), which corresponded to the ‘CW’ and ‘CCW’ categories, illustrating within-category transfer [7,8]. Furthermore, there was a correlation between estimation accuracy and discrimination accuracy in the data and between posttest-pretest changes in discrimination accuracy and the separation between pretest and posttest estimation distributions (AUROC values).

The PL effects we observed share some similarities with shorter-timescale effects of attention and adaptation. Specifically, spatial attention has been shown to improve behavioral performance by enhancing task-relevant sensory attributes [71,72], even when this leads to a ‘less veridical’ representation of the stimulus ([73]; reviewed in [81]). On slightly longer timescales, adaptation enhances direction discrimination for directions near that of the adaptor, while repelling perceived directions away from the adaptor [74,75]. Both these effects are relatively short-lived and therefore do not account for the results of the current study, which accumulated over multiple days.

Indeed, we found that the magnitude of bias did not increase over the initial training trials, as would be expected if adaptation primarily modified estimates (see Appendix A in S1 Text). In addition, a previous PL study examined a similar protocol with randomly intermixed leftward or rightward motion directions, thereby preventing adaptation, and found overestimation following training [7]. In the current study, given that ‘CW’ and ‘CCW’ motion directions for each of the three positive and negative deviations off the horizontal axis were also randomly intermixed, had adaptation affected estimates, the effect should have ‘canceled out’ across trials (i.e., adaptation to the ‘CW’ direction would be followed by adaptation to ‘CCW’). Moreover, had observers adapted to the horizontal direction (although it was never presented), we would have expected smaller estimation biases for motion directions further away from the horizontal (e.g., 8° versus 2°), but this was not observed (see Fig 1G). Most critically, the adaptation timescale cannot account for the differences between the training groups and the control group, given that pre-test and post-test sessions were identical for all groups. Thus, although short-term effects might have contributed partially to perception, they cannot explain the change in performance across days—the main finding of the study—which is driven by perceptual learning.

As in a number of previous studies, our experiments measure the effects of PL without feedback [45–49]. This approach aligns more closely with the “unsupervised learning” that occurs in many real-world situations. Moreover, several authors have suggested that it is preferable to avoid feedback when using estimation judgements to assess subjective appearance in the context of perceptual learning [7], category learning [76], interaction of subjective and objective perceptual organizations [77], 3-D form perception [78], and the appearance of various perceptual dimensions (e.g., [79,80]; review in [81]). In discrimination tasks in PL, biased feedback (i.e., reversed incorrect labels of categories) may shift the decision criterion, leading to subjective decision biases [81]. Future studies should examine methods for reducing estimation biases without using feedback that may bias reports of appearance.

PL studies typically use two-alternative choice tasks and only a few have used estimation tasks. Most of these provided feedback [82–84] and reported reduced estimation variability, but they did not examine shifts in estimate distribution (i.e., appearance). The only study using estimation in the absence of feedback found that estimation training increased overestimation in smooth eye movements and perceptual estimates [7]. Another PL study, which employed feedback during training, used both estimation and discrimination tasks and analyzed cross-task transfer [82]. Training on discrimination did not have a statistically detectable effect on estimation variance, and training on estimation did not affect discrimination thresholds. Within-task testing revealed enhanced discrimination performance and reduced estimation variance. These findings suggest that the absence of feedback was crucial for our findings of robust cross-task transfer, increased discrimination accuracy, reductions in discrimination thresholds, and persistent (non-reduced) estimation repulsions.

How did training lead to changes in the estimate distribution? To explain these findings, we developed a new model of the effects of perceptual learning on discrimination and estimation. We fit the model to the discrimination accuracies as well as the *entire* distribution of estimates, PRE- and post- training, allowing us to quantitatively account for non-standard forms of variability within and between participants. Our model combines encoding and decoding components: (1) efficient encoding of cardinal motion directions —where more resources are devoted to directions that are more common in the natural environment; (2) conditional inference—where observers implicitly categorize motion and then condition their estimates on their category decision; and (3) gain modulation–increased precision of representation for trained motions in both tasks. We demonstrate that the combination of these components allows the model to explain overestimation and bimodality of estimates pre-training, as well as the changes in these estimates post-training.

The first two model components are necessary to explain the pattern of estimate distributions observed at pre-test (e.g., the bimodality of the estimate distribution, the magnitude of overestimation, and misclassified estimates far from the boundary). The magnitude of overestimations we found before training are similar to that in other studies [30,50,51]. The first component in the model is efficient encoding, which warps the tuning curves around the horizontal such that more resources are devoted to near-horizontal directions because they are more commonly encountered in the environment [67] Fig 2A). We show that this component enables the model to capture the pre-training estimate distributions. This effect and other seemingly anti-Bayesian percepts can be explained by computational models using efficient encoding [34,70]. Although priors are often associated with attractive biases, in the case of efficient encoding the prior can also lead to repulsive biases, as seen in the oblique effect (i.e., high sensitivity and yet high bias near the cardinals). When efficient encoding leads to asymmetric tuning curves with heavier tails on the side of lower prior density, repulsive bias can emerge (Fig 2A).

In the current study, the extent of the bias and the bimodal shape of the distribution suggest that a second component is also at play–implicit categorization. Explicit categorization before estimation judgments has been shown to lead to bias and bimodality in estimate distributions [33,50,51]. Note that we observe clear bimodality in our data, as also seen in the Jazayeri and Movshon study ([51], Fig 1). However, their analysis was based on the mean of estimation bias for correctly decided trials (Fig 2e). Our computational model aims to explain the full estimation distribution, using the hypothesis of “conditioned perception”—estimation is based on sensory evidence but conditioned on a category decision rather than across all possibilities [33,50]. Fig J in S1 Text shows a version of our model without conditioned perception, which fails to capture the bimodality of the estimated distributions.

The implicit categorization component of our model is agnostic as to whether this ‘decision’ is unconscious or conscious (i.e., observers consciously try to make estimates that are consistent with the category they would have chosen given the noise in the stimuli). In a prior study with intermixed horizontal and near-horizontal directions, there were similar estimation biases for the near-horizontal directions (e.g., mean estimates of ~ 9° for 3° motion direction; [7]). This finding suggests that estimation biases may emerge even when observers see and estimate horizontal motion and thus are incentivized to not avoid the boundary. Regardless, implicit categorization, which could also be interpreted as a “post-perceptual” process, interacts with the sensory components to produce behavior—a topic worthy of further investigation. Some evidence indicates that early sensory neurons are impacted not only by the sensory input itself but also by decision-related components, illustrating that that perceptual decision-making is not a mere feedforward mechanism, but includes top-down processes that can influence neuronal gain [85,86]. In the same vein, in some computational models, early sensory processing depends on contextual modulation, such as related expectations [87,88].

Why would observers perform an implicit categorization in the estimation task when they were not instructed to do so? Empirical [89,90] and theoretical [52,91] studies suggest that observers may implicitly commit to a high-level interpretation of a stimulus before estimating it (i.e., conditional inference). This process has been considered a perceptual analogue of confirmation bias [92]. A recent study and model also proposed that continuous feedback about category certainty from decision to sensory areas reflects a form of perceptual confirmation bias on short time scales [93]. Although such biases may seem detrimental to perception, conditional inference may confer distinct advantages [52], including reducing energy costs [94,95], optimizing the use of neural resources by discarding unnecessary details about a stimulus [33], and protecting crucial information from internally generated noise by storing it in a discrete format [52]. Specifically, such a strategy is advantageous when the observer has a good chance of correctly discriminating a stimulus in the presence of post-decisional noise [50,52].

Why do observers implicitly categorize around *horizontal*? One explanation is that observers were influenced by the experimental design, either because stimuli were symmetrically distributed around the horizontal boundary or because they were asked to perform the discrimination task during the pre-test (albeit in separate blocks of trials). Another possibility is that clockwise and counterclockwise motions off horizontal motion are “natural categories” [29] shaped by the structure of the world (i.e., near-cardinal motion is more common than near-oblique motion). Because observers exhibit repulsive biases away from cardinals (both clockwise and counterclockwise), these two groups of percepts clump together over years of experience, forming internal categories that are then used to make perceptual decisions.

A real-world example of this is that of hanging a picture on a wall—very small amounts of off-axis tilt are quite noticeable. Indeed, the use of horizontal as an implicit reference, even when it is not explicitly presented, has been suggested previously [96]. Moreover, the need for a perceptual categorization process to predict anisotropies in motion direction perception, repulsion in estimates from the horizontal, *and* high sensitivity near the horizontal has been suggested in computational modeling work [97], although how and at what stage this categorization step takes place remains unclear. The emergence of ‘natural’ category boundaries has been recently demonstrated in neural networks trained on objects in natural images, which exhibit biases consistent with human category boundaries in color perception [98]. These examples provide an intuition for the relation between high sensitivity around ‘anchor‘ stimuli and the grouping of nearby values into internal categories.

The third component in the model, gain modulation, was needed to explain how behavioral results changed with training–improved discrimination accuracy and multiple changes in the estimate distribution. We modeled PL as a change in the gain of sensory neurons representing the trained motion direction. Explanations of the mechanisms underlying PL range from low-level changes in sensory neurons—e.g., changes in gain [16,23] or tuning [12,14], feed-forward weights, and noise correlations [24]—to top-down modulations of neural responses based on context and task [17,18,57], changes in decision-making [13], and reweighting [5,26]. Different neuronal changes may underlie performance improvement in PL. A human fMRI study demonstrated that PL may strengthen attentional (gain) modulation of sensory representations in the cortex [23], an account that has been supported by physiological recordings in cat visual cortex [16] and gerbil auditory cortex [69]. Tuning changes in sensory neurons (i.e., sharpening and lateral shifts in individual tuning curves) may also be important for PL [12,14,17,57]. Accordingly, we fit a variant of our model in which we replaced gain modulation with tuning changes (see Fig J in S1 Text), such that neurons encoding the trained stimuli would be more densely packed after training stimuli (motion directions) [67]. This model variant reproduced human behavior nearly as well as the gain-change model, consistent with the fact that various neural mechanisms may explain the observed behavior. Our behavioral data are not sufficient to conclusively adjudicate between these possibilities. We speculate that both model variants, changes to sensory neurons in gain or tuning, may also potentially account for the decrease in coherence thresholds observed in the data after learning, although we did not model coherence thresholds. Whether gain or tuning, our model and data illustrate that a simple change in a sensory neural representation may interact with implicit categorization to produce idiosyncratic and unexpected patterns of behavior with learning.

An intriguing aspect of our findings is that PL concurrently reduced noise coherence thresholds and shifted estimate distributions. Some previous models and studies of estimation bias [34,50,51,70] predict that estimation biases should decrease as sensory noise decreases (e.g., controlled by the motion coherence of a RDK [50,51]. In contrast, in our study, although the noise coherence thresholds *decreased* after training (a known consequence of perceptual learning, (e.g., [54–58]), estimate distribution shifted further away from horizontal than at pre-test. This aspect of the data does not appear to be predicted by previous computational models [34,50,70]. Our model illustrates how increased precision can account for the coupling between increased estimation biases and increased sensitivity after learning. By linking efficient encoding, conditional inference, and gain or tuning changes, our model suggests that the perceptual system may use different mechanisms in tandem to represent and interpret information more effectively and better tune to ongoing changes in task and environment.

Theories of efficient coding may offer a unifying framework relating PL, discriminability, and perceptual biases. The brain allocates more resources to representing features of the environment that are more common. This efficient allocation of resources must occur through some sort of learning process throughout development—which may well be a form of PL. For example, cardinal orientations (horizontal/vertical) are more common than oblique orientations in natural and retinal images due to the aligning influence of gravity on the orientation of environmental structures and visual observers [65]. Correspondingly, the brain devotes more sensory neurons to the cardinal orientations, and human observers exhibit better discriminability around the cardinals than obliques, but also larger biases away from them [29–32]. This is true for many sensory features, including motion direction, and theories of efficient coding can successfully predict discriminability and estimation biases in human and animal behavior based on environmental statistics [34,65–67,70].

To account for biases in motion perception that were even present on day 1, the model assumes that the brain devotes more sensory neurons to representing horizontal motion (component 1 of the model). That is, tuning changes probably occurred over development due to exposure to non-uniform environmental statistics. Our model variant that employs training-induced tuning changes could have a similar interpretation but over a shorter timescale. This model variant assumes that the same sort of environmentally-driven tuning changes that occur across development may also happen across training days in the experiment—with increased tuning for the specific stimuli presented during training. This model variant can predict the behavior in our study before and after learning. Thus, it may be that a similar mechanism of efficient coding underlies PL, whether in development or adulthood, which enhances discriminability at the potential cost of maintaining or even increasing perceptual biases. Our empirical findings and observer model show that PL-induced increases in the precision of sensory encoding can interact with implicit categorization and a non-uniform internal representation to repel percepts away from a discrimination boundary. Interestingly, repulsion in the appearance of near-boundary stimuli resembles a well-known characteristic of category learning–between-category expansion–the repulsive distortions of values near the category boundary [99].

Category learning typically enhances discrimination between categories, whereas within-category discrimination is reduced or remains unchanged. For example, in color perception, an object belonging to a group of mostly red objects is judged to be redder than an identically-colored object belonging to another group of mostly violet objects [76]. That is, color appearance is distorted toward category means, based on the hue statistics of the two groups. Despite some findings of associations between category learning and PL [8,9,82], effects of category learning on appearance (e.g., between-category expansion) had not been explored in PL. Our model strengthens the links between category learning and PL [8,9,100] and demonstrates that distinctions between perceptual categories may be enhanced by changes in sensory encoding (e.g., in gain) over the course of training, without necessarily needing to invoke additional changes in cognitive or decision-making processes.

The present findings can have translational implications for real-world manifestations of PL such as perceptual expertise and clinical rehabilitation. For example, when learning to categorize CT images into “cancerous” and “benign” [44,101,102], a radiologist becomes increasingly sensitive to differences between similar images, more accurate, and better at her job. The discriminating features become increasingly salient over training, altering the appearance of both cancerous and benign images such that they appear more dissimilar from each other. Moreover, our results should also be taken into account when developing protocols and assessment of perceptual rehabilitation of special populations, such as people with amblyopia [37–39] and cortical blindness [40–43].

In conclusion, we have shown that PL improves discrimination but not appearance, and that these effects can be ascribed to increases in the coding precision of the trained stimuli.

## Methods

### Ethics statement

Approval for this study was granted by the New York University committee called – “University Committee on Activities Involving Human Subjects” (approval number IRB-FY2016–466).

### Observers

Twenty-three human adults participated (mean age = 28.9, SD = 1.9; nine males). All observers provided written informed consent under the University Committee’s protocol on Activities Involving Human Subjects at New York University. All had normal or corrected to normal vision, were untrained and did not know the experiment’s purpose. Two participants did not meet the criterion during pre-test to be enrolled in the experiment: One could not perform the estimation task (average estimation deviated more than 70º from veridical); the other could not perform the discrimination task above chance. Thus, twenty-one observers participated in the study. Data is available at https://osf.io/mep9v/?view_only=c97fc183184042c48e45c8a000c793c5.

### Visual stimuli and display

Stimuli were random dot kinematograms (RDK) with dots moving at 15º/s in a stationary aperture with a 5º radius, sparing 0.75º around the central fixation cross. On each frame each dot was assigned a direction that was either the coherent direction or a different random direction according to the coherence level found for each observer, noise dots moved randomly across directions (i.e., directional noise; Brownian motion) and dots were warped when they moved out of the aperture [53]. On each frame 5% of the dots were redrawn to a new position in the aperture to limit tracking of individual dots. Dots were black (4 pixels, 3 cd/m2) and were shown on a uniform gray background, with dot density 1.65 dots per square degree. No horizontal reference line was presented to participants. Stimuli were displayed on a calibrated 41x30 cm CRT monitor (IBM P260) with a resolution of 1280x960 pixels, and a 100 Hz refresh rate. Observers were seated at 57 cm distance from the screen with their head supported by a combined chin- and forehead-rest.

### Testing and training tasks

The experiment consisted of five 60-min sessions, one per day, over five consecutive days; the first (pre-test) and last (post-test) sessions were identical, and the three intermediate sessions were training sessions (Fig A). The pre-test included a short practice on both the estimation and the discrimination tasks. For each observer we tested discrimination coherence thresholds for motion directions of ± 4º using three randomly interleaved 60-trial 3-down-1-up staircases. We estimated coherence thresholds by averaging the thresholds reached by the three staircases. This coherence level was then used for all following testing and training.

During PRE- and post-test sessions, we measured performance on six randomly presented coherent motion directions (directions relative to horizontal to the right): -8º, -4º, -2º (downwards from horizontal) and 8º, 4º, 2º (upwards from horizontal). Testing sessions (pre-test and post-test) included a block of motion discrimination and a block of direction estimation; the order of the two blocks was counterbalanced across observers. Each block consisted of 360 trials. In training sessions, observers were trained either on the estimation task or in the discrimination task, only directions of ± 4º directions were presented, and each session consisted of 720 trials presented in four blocks. For the control group, no training was provided, as is the case in some PL studies [103–105].

Each trial started with a 500-ms fixation cross at the center of the screen, then the 200-ms RDK appeared followed by a 700-ms ISI after which an auditory start signal indicated that a response could be given (Fig 1A). In discrimination blocks, observers pressed a key on the keyboard indicating upward or downward motion relative to horizontal, and the response had to be given within 900 ms. In estimation blocks, a randomly oriented line appeared and, using a mouse, observers were given 4-seconds to orient the line according to the motion direction they perceived; the initial orientation of the line varied uniformly around horizontal with a variance of 5º. Observers mostly responded within the timeline (98% of trials). No feedback was given either for discrimination or estimation tasks as feedback is not necessary for PL to occur and could affect reports rather than appearance (see Discussion).

#### Statistical analysis methods.

For both estimation and discrimination tasks, up and down responses were combined: we negated directions and responses for the downward trials and merged the data with that of the upward trials. We chose this approach also for the estimation task, rather than examining the absolute estimation values, because the negative values of estimations (i.e., to the incorrect side; for a presented direction of 4º a response of -6º) would be considered as relatively ‘correct responses’ (i.e., 6º, or only +2º bias, whereas the original response was -8º bias).

#### Mixed design ANOVA.

We used a repeated measures analysis of variance (ANOVA), using session (pre-test/post-test) and directions (±2º, ± 4º or ± 8º) as within-subject factors, and group or training conditions as between-subject factors. When assumptions for sphericity were not met, results were corrected using Greenhouse-Geisser. Mixed design anova was used to analyze coherence thresholds, discrimination accuracy, signed estimation accuracy, means and modes of the estimate distributions.

#### Cluster-mass permutation tests.

To examine changes in the estimate distribution without assuming a particular shape for the distribution (given that for some participants the distribution was bimodal), we used permutation based cluster mass tests [59] used to examine significant clusters in previous studies [106–109]. This allowed us to detect non-parametric shifts in the estimate distributions from pre-test to post-test. First, for each participant, estimation data were collapsed across negative and positive directions (-2º were collapsed with the + 2º) and across directions (2º, 4º, and 8º). Motion directions were then binned in 5º bins (for pre-test and for post-test) between -70º to + 70º (0º being horizontal) to create counts of estimates in each motion direction bin for the pre-test and post-test.

In step 1 of the analysis, for each group we used a Poisson generalized linear mixed model to examine whether the number of estimates significantly differed between pre-test and post-test in that motion direction bin across participants in the group (i.e., nEstimates ~ Session + (1 | subj)). A bin was considered significant if the z-score was above or below the critical z-values in the sampling distribution of the permuted z-scores (see random permutation in step 2; two-tailed, alpha = 0.05). In step 2, we identified clusters of adjacent significant estimate bins with an effect in the same direction (pre>post or post>PRE). The clusters that emerged were considered significant by comparing the summed z-score value of the cluster to the distribution of summed z-score values from clusters derived from 1000 random permutations of the data.

To create permuted data, the PRE/post labels were swapped within, but not across, participants. Labels were swapped for that participant across all estimate bins. For each permutation, step 1 and 2 were repeated to extract the largest cluster in that permutation. This process led to a distribution of clusters (i.e., of largest summed z-scores across 1000 random permutations). Finally, the original cluster that emerged in data was considered significant if clusters with same or larger values occurred in less than 5% of the randomly permuted datasets (two-tailed values may either be positive or negative depending on whether pre-test was larger than post-test or the opposite). This process was done for each training group and a similar process was used to examine significant clusters in the interaction between training groups and control and session (i.e., nEstimates ~ Group * Session + (1 | subj)). Given that the number of estimates is fixed, a shift between pre-test and post-test, would likely mean that if a significant cluster emerged where post-test > prettest, there would likely also be a pre-test>post-test cluster in the estimate distribution. Thus, we also illustrated the results for the marginally significant clusters of the randomly permuted datasets but opposite sign (alpha = 0.1, two tailed; Fig 1D).

#### Permutation-based AUROC test.

To measure shifts in the estimate distribution with PL, we conducted an AUROC analysis [60]. For each observer and stimulus motion direction separately, we measured the area under the ROC curve (AUROC) between the PRE- and post-test distributions of correctly-classified estimates. This non-parametric analysis quantifies separation between the distributions; e.g., a value of 0.5 indicates complete overlap, whereas a value of 0 or 1 indicates a complete shift/separation in either direction. The reason we analyzed only the correctly-classified estimates in this analysis is because we wanted the metric to be independent of our measure of signed estimation accuracy, (i.e., not influenced by changes in the relative density of the multi modal distribution on each side of the discrimination boundary). This is also enforced because AUROC is based on the respective cumulative distributions PRE- and post-test. AUROC can capture a variety of non-parametric shifts even in strangely shaped distributions, including changes in tail thickness, so it is well-suited to the multi-modal and skewed estimated distributions we observed. For the group level statistics, we tested the AUROC values for each group across all directions either between groups (training versus control; independent samples t-test), or for each group against the value of 0.5 (i.e., complete overlap between PRE- and post-test distributions) to examine whether the distributions changed between sessions. For the individual level statistics, we conducted a two-tailed permutation test for each observer and stimulus direction individually, then performed multiple comparisons correction. For the permutation test, we swapped the pre-post labels 1000 times to construct a null distribution of AUROC scores and then computed the two-tailed p-value. False discovery rate was controlled for using the method of Benjamini, Krieger, and Yekutieli (2006) [110]. Only comparisons p-values surviving the correction were marked as significant, and we mark that observer with an asterisk only if all three directions for a specific observer were significant (Fig D in S1 Text).

#### Modeling methods.

We hypothesized that PL in our task can be explained by increases in the precision of the internal sensory representation — a representation that is already warped to devote more resources to certain features. To test this hypothesis, we created a probabilistic observer model that formalizes each of its commitments. Probabilistic observer models (e.g., Bayesian observer models) are most often used to describe how an observer should behave in a task to optimize their performance, given some variability (e.g., uncertainty, ambiguity, sensory noise) that interferes with decision-making [111,112]. Such models rely on the assumption that the observer (or some part of their brain) is “aware” of these noise sources, e.g., the distribution of a stimulus’s value across trials, the brain’s noisy measurement of the stimulus, and so on [68]. However, it is straightforward to modify the model such that the observer has some set of incomplete or incorrect beliefs [113,114]. These “imperfectly optimal observers” [115] can reproduce idiosyncrasies in human behavior while maintaining the fundamental commitment that observers account for their own sensory uncertainty in decision-making rather than ignoring it. Such observer models can account for complexities in human behavior, without being beholden to explaining how each step is part of a normative account or optimal solution. We propose one such observer model, consisting of encoding and decoding stages, which “learns” and performs both the discrimination and estimation tasks. The code for the computational model will be available online: https://github.com/csb0.

#### Task structure.

On every trial, there is a 0.5 probability of the stimulus motion direction being CW of horizontal, expressed as *p(C* *=* *CW)* = 0.5, where *C*, the category of the stimulus, takes the value CCW or CW. The overall stimulus distribution, *p(s),* is the normalized sum of six delta functions at ± 2, ± 4, and ± 8º, i.e., the probability of each of the six possible motion directions is equal. Once the category is drawn on each trial, the new, “category-conditioned” stimulus distribution *p(s | C)* is the half of *p(s)* on the side of the discrimination boundary (0º) that is consistent with the category *C*.

#### Encoding.

We assume the stimulus is measured (“encoded”) by a population of *n* = 10 ‘neurons’ whose tuning curves tile the space of motion directions but are warped such that more neurons represent motion directions near the horizontal, consistent with physiological measurements and theoretical studies of efficient encoding [64,67]. The tuning curves specify each neuron’s mean firing rate as a function of stimulus direction. It has been demonstrated that the tuning widths matter more than the number of neurons sampled in these types of efficient encoding model [64,67]. Each tuning curve, before warping, is a single cycle of a cos^2 function raised to a power (0.3745) such that its full-width at half maximum (FWHM), *w*_*t*_, is 71º [67], consistent with typical tuning of motion-direction-selective neurons in macaque area MT [116]. Each neuron has a baseline firing rate, *b*, and a gain, *g,* which specifies the above-baseline firing rate for the preferred stimulus (i.e., the maximum response). The pre-training gains, *g*_*pre*_, are assumed to be identical across the population. The post-training gains are elevated according to a Gaussian profile across the population, centered at 0º, with a standard deviation equal to the spacing between neurons (specifically, with ten neurons, the standard deviation is 4º). A parameter *g*_*post*_ specified the gain of the most responsive neuron in the population. This captures our assumption that learning modulates the gain of sensory neurons, relative to their sensitivity to the trained motion directions.

Warping in our model embodies the efficient coding notion of devoting more neurons and spikes to more common stimuli [64,67]. This leads to a behavior that resembles avoidance of the boundary when estimating the stimulus, so we refer to this as the efficient encoding/ “boundary avoidance” component of the model. The neural population is formed from a set homogeneous (“convolutional”) set of tuning curves that are re-mapped by warping the motion direction axis according to a parametric function that is fit to the data of each observer, but assumed unchanged throughout the experiment (i.e., unaffected by learning). The warping function is defined as the cumulative integral of a cell “density” function: *h(s,a,w*_*b*_*,σ*_*b*_*) = a* + 2 + ɸ(*s,-w*_*b*_*/2,*σ_*b*_) *+* ɸ(*-s,w*_*b*_*/2,*σ_*b*_), where ɸ is the cumulative Gaussian function. Motion direction is designated with variable *s*, and the shape of the function is governed by three parameters: an amplitude, *a*, that controls the overall magnitude of the warping (i.e., how “extremely” the observer avoids the boundary); *w*_*b*_, the width of the region over which tuning curves are drawn toward the boundary; and *σ*_*b*_, the standard deviation of the cumulative Gaussians, which controls the fuzziness of the template’s edges, leading to warping that is more graded or discrete. Examples are shown in Fig L in S1 Text. Before use, the function *h* is numerically normalized to integrate to 1.

For a stimulus direction *s*, each neuron’s spike count *r*_*i*_, is drawn independently from a Poisson distribution, with rate determined by its tuning curve evaluated at the (warped) value of *s*. The noisy “population response” (i.e., the vector of spike counts from each neuron), denoted **r**, is a sample of the “measurement distribution” *p(****r**** | s)*.

#### Decoding.

The internal representation of the stimulus **r** must be “read out” or decoded to discriminate or estimate motion directions. For this purpose, we used a *maximum a posteriori* or MAP decision rule for the discrimination judgment, and Bayes-least-squares rule for the estimation task. Our methods are identical to those reported in other studies [61,117] so we refer the reader to their methods for details, and simply provide an intuition here.

Both decoders rely on a likelihood function, in which the measurement distribution is expressed as a function of the stimulus, *s*, for each noisy population response ***r.*** For our model, the log likelihood is equal to the sum (across neurons) of the log of each tuning curve, each weighted by the observed spike count of the associated neuron [61,117]. We incorporate one additional assumption, that the decoder is ‘unaware’ of the warping in the encoder, and so assumes a homogeneous (“convolutional”) encoding population in computing the likelihoods. This idea has been used successfully in the domain of adaptation to explain biases in perception [68].

The likelihood fluctuates randomly on each trial, due to the variability of **r**. In the case when *w*_*b*_ or *a* is zero, the likelihood is symmetric and centered on the true stimulus motion direction, i.e., its mean is an unbiased estimator of the true stimulus (equivalent to probabilistic population coding; [61,117]. Its width, which corresponds with the observer’s uncertainty about the motion direction generating the internal measurement **r**, depends on gain and baseline firing rate. If *w*_*b*_ and *a* are larger than zero, the likelihood is stretched away symmetrically in half around the boundary a distance depending on *w*_*b*_, and suppressed near the boundary an amount depending on *a.* The likelihood is therefore asymmetric, and the shape of its tails depends on *σ*_*b*_.

In the discrimination task, the decision variable *d* represents the log posterior ratio, and is determined by the proportion of the likelihood function that falls on each side of the discrimination boundary: If most of the likelihood mass falls on the positive side of the boundary, *d* > 0, the observer reports “CW” (and vice versa). The distribution of discrimination responses across trials is *p(C*_*est*_* | C)* and the discrimination accuracy *p(Correct)* = *p(d > 0 | C = CW)/2 *+* p(d < 0 | C = CCW)/2*. *C*_*est*_ denotes the estimated category. Note that *d* inherits its variability from the population response **r**. To model lapses in the discrimination judgement, we multiply *p(Correct)* by the same factor *1-*λ for all stimulus motion directions. Note that, in our model fitting, *λ* is never allowed to go below 0.5.

In the estimation task, the estimate is computed as the expected value of the posterior distribution, i.e., the Bayes-least-squares estimate [33]. The posterior is computed on each trial by imposing an implicit discrimination judgment using the rule described above, using the response (CW or CCW) as a conditional prior, *p(s | C*_*est*_), which has a value of 1 on the side of the discrimination boundary corresponding to the chosen category, and 0 on the other side (i.e., uniform across the chosen category of motion directions). The product of this conditional prior *p(s | C*_*est*_), and the likelihood *p(****r**** | s)*, is the posterior *p(s | ****r****,C*_*est*_), and the estimation response on a single trial is the mean of this posterior. The distribution of estimates across trials *p(s*_*est*_* | s)* is computed numerically for each stimulus, ± 2, 4, and 8º, via Monte Carlo simulation. To model motor noise in the execution of the response (orienting an arrow with a mouse), i.i.d. Gaussian noise with standard deviation *σ*_*m*_ is added to each estimate distribution.

#### Parameter estimation.

The model has six parameters that are fit to individual observers: *a, w*_*b*_*, σ*_*b*_*, g*_*pre*_*, g*_*post*_*, λ,* and five parameters that are shared across observers *b, w*_*t*_*, N, *σ_*m*_*, and *σ_*g*_. We estimated the individual parameters by minimizing a loss function L(**ϴ**) (with **ϴ** a vector containing the parameters) expressing the fit between each observer’s data and model behavior. L(**ϴ**) was defined as the weighted sum of two terms: (1) absolute difference (L1 norm) between the discrimination accuracy in the data vs. model behavior (i.e., sum of L1 differences across stimuli and PRE- vs. post-learning); and (2) the energy distance [118] between the estimate distributions for the data vs. model (sum of distances across stimuli and PRE- vs. post-learning). This distance metric takes into account the entire shape of the estimate distribution and is appropriate for comparing distributions with complex shapes (i.e., like the bimodal distributions we observed). We computed the energy distance as implemented in the Information Theoretic Estimators Toolbox in Matlab [119]. The weights on the two terms were used to rescale the two terms in the loss function into a similar numerical range. These weights were computed by evaluating the objective function on an initial random set of parameters, as is common practice in multi-objective optimization [120]. The loss function was stochastic, varying between each run of simulated trials due to sampling variability in the Poisson spike generation. We set the number of simulated trials to 1500 and repeated the optimization 10 times with a new set of initial random parameters.

We fit all free parameters simultaneously for each observer separately using an optimization algorithm designed for stochastic objective functions [121]. We ran it 10 times per observer, chose the iteration with the lowest loss. The resulting best-fit parameters were then used to evaluate the model using 1500 simulated trials, generating (stochastic) model behavior for the estimation and discrimination tasks (Figs 2B,C and 3).

Note that, for each observer, we fit the 6 free parameters with approximately 486 data points (80 estimation responses x 2 PRE/post-training x 3 motion directions, plus 1 discrimination accuracy x 2 PRE/post-training x 3 motion directions), so this is a well-constrained optimization problem (as compared to fitting only the estimation biases for the 6 stimulus directions).

#### Model comparison.

We compared models using iterated *k*-fold (*k *= 3) cross-validated loss. For each observer and each model, we averaged the CV-loss across folds and iterations (folds x iterations = 102), subtracted this from the average CV-loss from the full model, and then averaged this across observers to quantify each model’s generalizability relative to the full model (Fig K in S1 Text). We also did the same with the average in-sample (training set) loss across observers to quantify goodness-of-fit relative to the full model. To perform the cross-validated model fitting, for each observer, we computed one normalization factor for each objective (estimation and discrimination) based on a fixed initial set of parameters and kept these normalization factors fixed across models. Note that these factors were quite similar across observers, but not identical. This was essential to keep the training and test losses in the same space across models and hence comparable.

## Supporting information

S1 Text Fig A. Mixture model results. Fig B. QQ-Plots of percentile values of estimate distributions at pre-test versus post-test. Fig C. Individual participant data. Fig D. Auroc results. Fig E. Model and Data performance. Fig F. Human behavior vs model behavior for control (no training) observers. Fig G. No-gain-change (“no GC”) model behavior. Fig H. Variant of full model (gain modulation replaced with efficient coding tuning changes), “TC”. Fig I. No-boundary avoidance (“no BA”) model behavior. Fig J. No-conditional-inference (“no CI”) model behavior. Fig K. Model comparison. Fig L. Example boundary avoidance templates, for different values of *w*_*b*_ and *σ*_*b*_. Fig M. Efficient coding/ tuning changes model, “TC_reduced”. (DOCX)
