# Supplementary material for: Perceptual learning improves discrimination but does not reduce distortions in appearance
Source: PLoS Comput Biol. 2025 Apr 15;21(4):e1012980. doi: 10.1371/journal.pcbi.1012980 (PMC12047783; doi:10.1371/journal.pcbi.1012980)
Supplement: S1 Text — (DOCX) [file pcbi.1012980.s001.docx]

**Supporting Information**

**Appendix A**

**Reaction times.**

Although our main measures were estimation performance and discrimination accuracy, we also analyzed reaction time (RT) on correct trials and found no speed accuracy trade-offs: reaction time decreased between pre-test and post-test sessions (Estimation: F (1,18)=12.88, p=.002; Discrimination: F (1,18)=6.16,p=.02) but not differentially between training groups (interaction session X direction X training task, Estimation: F (4,36)=1.77, p=.16; Discrimination: F (4,36)<1).

**Ruling out adaptation: Estimation responses did not systematically increase over the first few trials of the training**

To rule out the possibility that adaptation accounts for the large estimation biases we observed, we analyzed the first 50 trials of the training for the estimation task. If adaptation underlies the large estimation biases we observed, absolute estimation biases should increase over the course of the first few trials of the experiment. For this analysis, we define estimation bias as the difference between the estimate response and the value of the stimulus presented (e.g., for a stimulus value of 2º and an estimation response of 5º the estimation bias would be 3º). To test this possibility of adaptation, we fit linear regression models to the first 50 trials of the training (session 2) of the group that trained on the estimation task (n = 7) and the trained stimulus direction ( -4º and +4º). We found that the mean absolute best-fit slopes (grand mean, 0.26º) — i.e., the change in absolute estimation response per trial —did not differ from flat slopes expected under the null hypothesis (grand mean, 0.005º). That is, we could not reject the null hypothesis that the slopes we observed were greater than what would be expected from sampling error alone. We obtained this result using 14 one-tailed permutation tests (14 tests – for n=7 participants and 2 directions per participant), wherein for each stimulus and observer, we shuffled the first 50 estimates for that stimulus and then computed a null slope, repeated this 1000 times, and compared the observed best-fit slope to the null distribution. 0/14 tests were significant with a corrected alpha of 0.0036 following Bonferroni correction. This demonstrates that the estimation responses did not increase with training due to adaptation.

Note that the stimuli (-4º and +4º,) were randomly interleaved across training trials, which would presumably prevent neural adaptation at the timescale of individual trials (as is typically used in experimental manipulations of adaptation). Also, the mean absolute estimation bias on the very first trial in the experiment across participants was large - a bias away from ±2°of 1.8º (i.e., mean estimate of 3.8º), from ±4 of 7.7º (i.e., mean estimate of 11.7º), and from ±8º of 8.8º (i.e., mean estimate of 16.8º). This means that participants came into day 1 of the experiment already with biases, consistent with our modelling assumption that the biases arise in part from observers’ individual neural tuning for motion direction.

**Appendix B**

**Reduced models**

To evaluate the effects of the primary model components (i.e., gain modulation, conditional inference, and efficient coding), we created three reduced models by removing model components one-by-one (**Fig. G, I and J** in S1 Text): (a) For the no-gain model (i.e., akin to the hypothesis for the control observers), we simply set the pre- and post-learning gain factors to be equal; (b) For the no-conditional-inference model, we computed the estimation response on each trial as the mean of the unmodified posterior *p (****r****|s)*; (c) For the no-efficient coding model, the parameters describing the boundary-avoidance decoding template, *f, a, and w_b_,* were all set to 0, with the consequence that the likelihood *p (****r****|s)* peaked at the true stimulus motion direction and was truncated based on the implicit discrimination judgment (i.e., similar to the models in refs [1–3].

**Tuning changes can explain human behavior nearly as well as gain modulation**

We modelled the effects of training as gain modulation of sensory neurons encoding the trained stimuli, consistent with the results of [4–6]. But there are other means by which encoding precision can be increased. For example, some have suggested that changes in neural tuning — i.e., changes in the density of neurons representing particular stimuli and changes in their widths — are important for PL [7–10]. Along these lines, we implemented two variants of our model, in which we assumed that exposure to the trained stimuli over the learning phase of the task causes a gradual increase in the number of neurons that prefer those stimuli. A similar learning mechanism has been derived from the theory of efficient coding, in which the distributions of sensory neuron preferences are matched to the environmental distribution of stimulus attributes such as visual orientation [11–14].

In the first model variant (“TC”), we took our full model (**Fig 2A**), and simply substituted tuning changes (training-driven efficient coding) for gain modulation, leaving the (environmental) efficient coding / boundary avoidance and conditional inference steps in the original model. Specifically, we assumed that training caused neurons around the trained stimuli to change in their density, according to the efficient coding solution given in ref [11]. These tuning changes act to maximize mutual information between the stimulus distribution and population response. This model variant reproduced each aspect of human behavior well, albeit with two more parameters than the full model (**Fig.** **H** in S1 Text). This model had comparable goodness-of-fit and generalization performance to the full model (**Fig. K** in S1 Text). For nearly all observers, the optimal encoder that best explained human behavior had a higher density of neurons around the trained stimuli (±4º motion) and a lower density further away (i.e., a more exaggerated version of the warping in the pre-training population). Note that this also corresponded to higher density of neurons representing the discrimination boundary at 0º, which corresponds with a training-related/contextual prior that is higher around the boundary and trained stimuli. These model comparisons demonstrate that training-dependent tuning changes in an already non-uniform sensory population, along with implicit categorization, can explain our data as well as the original model (which assumed that training modifies sensory gain). Thus, with our current data, we cannot conclusively adjudicate between these alternatives.

In the second model variant (“TC_reduced”), we tested how well such tuning changes, on their own, could account for the behavioral data. To do so, we simply removed the boundary avoidance and conditional inference components from the TC model. That is, we assumed that observers came into the experiment with a homogeneous population of sensory neurons representing motion direction, which was then modified by exposure to the training stimuli. This model captured increases in discrimination accuracy and estimation bias with learning, and their pattern of transfer across stimuli (to a lesser degree than the gain modulation model) but failed to capture the bimodality of the estimate distributions and the relations between discrimination accuracy and signed estimation accuracy. The estimation bias was under-predicted by the model and the estimation variance was over-predicted (**Fig. M** in S1 Text). Overall, this model had poor goodness-of-fit and generalization performance compared to the full model (**Fig. K** in S1 Text).

**References**

1. Stocker AA, Simoncelli EP. A Bayesian Model of Conditioned Perception. Adv Neural Inf Process Syst. 2008;20: 1409–1416.

2. Luu L, Stocker AA. Post-decision biases reveal a self-consistency principle in perceptual inference. eLife. 2018;7. doi:10.7554/eLife.33334

3. Qiu C, Luu L, Stocker AA. Benefits of Commitment in Hierarchical Inference. Psychol Rev. 2020 [cited 19 Jul 2021]. doi:10.1037/REV0000193

4. Byers A, Serences JT, Byers A., T. SJ. Exploring the relationship between perceptual learning and top-down attentional control. Vision Research Dec 1, 2012 pp. 30–39. doi:10.1016/j.visres.2012.07.008

5. Byers A, Serences JT. Enhanced attentional gain as a mechanism for generalized perceptual learning in human visual cortex. J Neurophysiol. 2014;112: 1217–1227. doi:10.1152/jn.00353.2014

6. Chen N, Bi TT, Zhou TT, Li S, Liu Z, Fang F. Sharpened cortical tuning and enhanced cortico-cortical communication contribute to the long-term neural mechanisms of visual motion perceptual learning. NeuroImage Academic Press Inc.; Jul 5, 2015 pp. 17–29. doi:10.1016/j.neuroimage.2015.04.041

7. Adab HZ, Popivanov ID, Vanduffel W, Vogels R. Perceptual learning of simple stimuli modifies stimulus representations in posterior inferior temporal cortex. J Cogn Neurosci. 2014;26: 2187–2200. doi:10.1162/jocn_a_00641

8. Li RW, Levi DM, Klein S a. Perceptual learning improves efficiency by re-tuning the decision “template” for position discrimination. Nat Neurosci. 2004;7: 178–183. doi:10.1038/nn1183

9. Schoups A, Vogels R, Qian N, Orban G. Practising orientation identification improves orientation coding in V1 neurons. Nature. 2001;412: 549–553. doi:10.1038/35087601

10. Yang T, Maunsell JHRR. The effect of perceptual learning on neuronal responses in monkey visual area V4. J Neurosci Off J Soc Neurosci. 2004;24: 1617–1626. doi:10.1523/JNEUROSCI.4442-03.2004

11. Ganguli D, Simoncelli EP. Efficient Sensory Encoding and Bayesian Inference with Heterogeneous Neural Populations. Neural Comput. 2014;26: 2103–2134. doi:10.1162/NECO_a_00638

12. Ganguli D, Simoncelli EP. Implicit encoding of prior probabilities in optimal neural populations. Adv Neural Inf Process Syst. 2010;2010: 658–666.

13. Wei XX, Stocker AA. Lawful relation between perceptual bias and discriminability. Proc Natl Acad Sci U S A. 2017;114: 10244–10249. doi:10.1073/pnas.1619153114

14. Wei X-X, Stocker AA. A Bayesian observer model constrained by efficient coding can explain “anti-Bayesian” percepts. Nat Neurosci. 2015;18: 1509–1517. doi:10.1038/nn.4105

**Supplementary Figures**


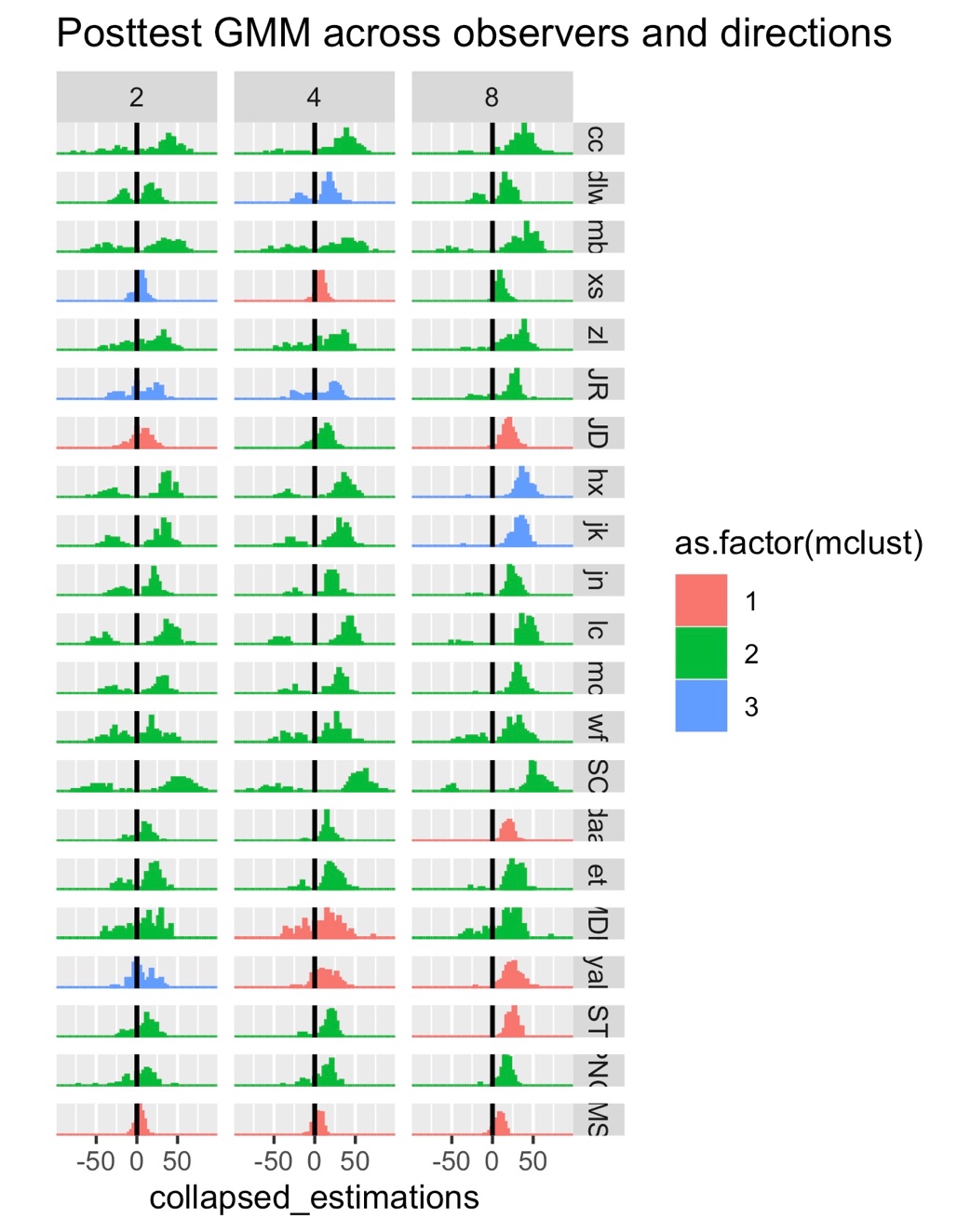

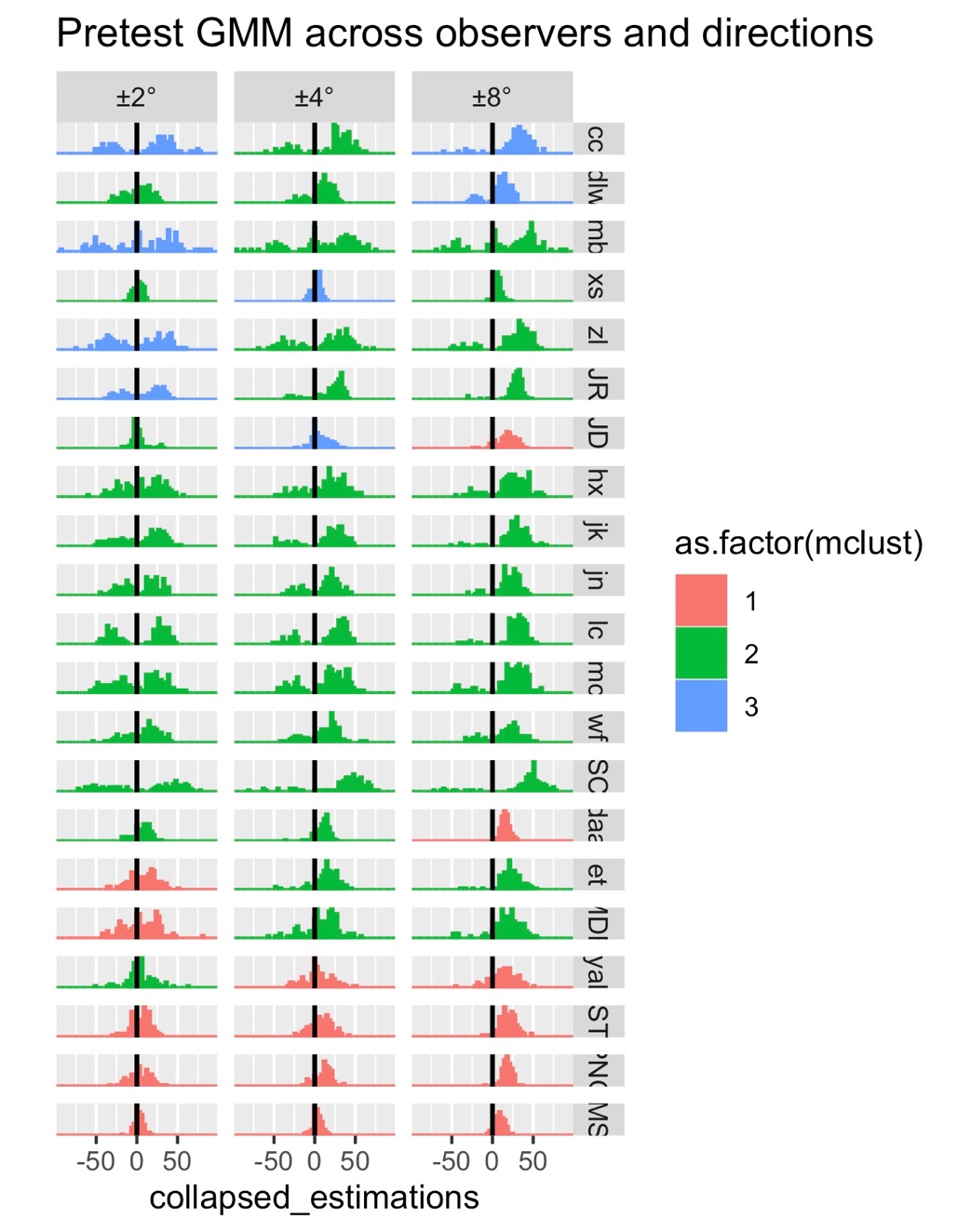

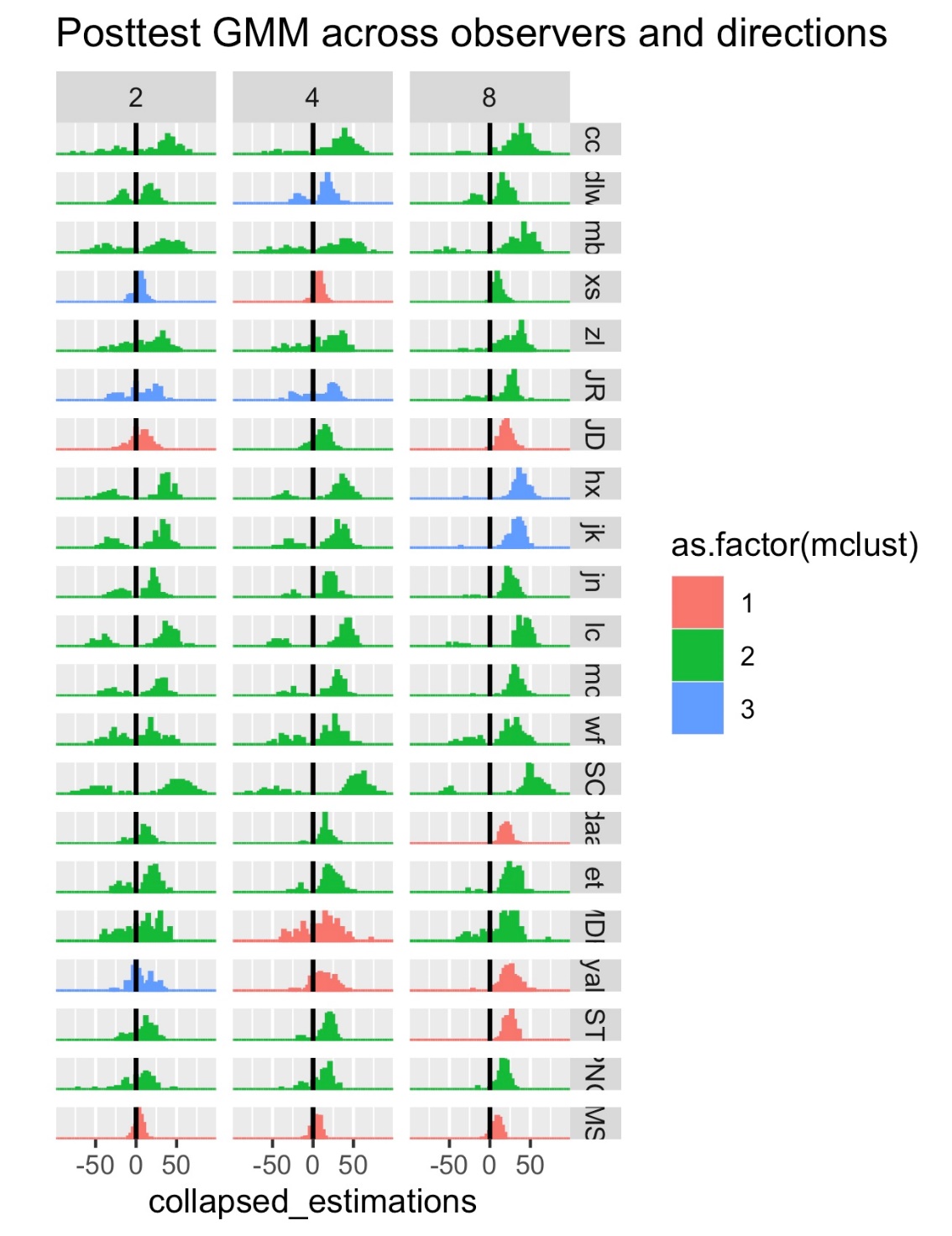


**Pretest**

**Posttest**

**Number of components:**

**Fig. A.** Mixture model results (used to assess level of bimodality) at pre-test and post-test across all observers (rows) and directions (columns).

**Fig. B.** QQ-Plots of percentile values of estimate distributions at pre-test versus post-test (percentile values between 0.05 and 0.95). X-axis- pre-test percentile values; Y-axis-post-test percentile values. Plots represent the means across participants in each group and direction (i.e., 2º, 4º, 8º).

**Fig. C.** (**A**) Violin plots for discrimination accuracy – each dot represents a participant and each row a motion direction (i.e., 2º, 4º, 8º) and lines connect pre-test to post-test value. (**B**) Violin plots for signed estimation accuracy, same format like A. (**C**) Pre-test versus post-test thresholds for each participant.

**Fig. D.** (**A**) AUROC values for each participant in the training groups (x-axis) and each motion direction (i.e., 2º, 4º, 8º). Y-axis represents difference in AUROC value before and after training. The test examines the overlap between pre-test and post-test distribution of correctly classified estimates (a value of 0.5 represents complete overlap). ‘Away from horizontal’ means that the distribution of estimates at post-test shifted to the ‘right’, away from pre-test and from horizontal; ‘towards horizontal’ means that the distribution of estimates at post-test shifted to the ‘left’, towards horizontal compared to pre-test; (**B**) AUROC values for control group, same format as in A. For the Training group, 7/14 participants showed a significant increase in AUROC after training for each of the three motion directions, and only 1/14 participant showed a significant decrease. In contrast, for the Control group, 2/7 observers showed a significant increase and 1/7 showed a significant decrease.

**A**

**C**

**B**

Post-test

**Fig. E. Model and Data performance. A.** Data and model fit from representative individual observers (O7, O12, O13, O15) from each training group. Same format as Fig. 2B-E. **B.** Correlation between signed estimation accuracy and discrimination accuracy in the model, collapsed across motion direction and training group for both testing sessions in pre-test (black circles) and post-test (red triangles). Same format as Fig. 1H. **C.** Pre- vs. post-test estimation mean; Top: Data; Bottom: Model. Same format as Fig. 3A-B.

**Fig. F. Human behavior vs model behavior for control (no training) observers.** Model reproduces the lack of PL in the control group in both (A) discrimination, (B) estimation discrimination tasks, a correlation between estimation accuracy and discrimination accuracy, and captures inter-observer variability.

**Fig. G. No-gain-change (“no GC") model behavior**. A variant of the full model without training-induced gain change in sensory neurons exhibits a lack of PL, but still shows a strong correlation between estimation and discrimination accuracy. Model fit to both pre- and post-training data, even though gain was fixed. For panels C-E, all trained observers included (in this and in all subsequent supplementary figures).

**Fig. H. Variant of full model (gain modulation replaced with efficient coding tuning changes), “TC”.** A variant of the full model, but with training-induced tuning changes instead of gain changes, shows similar behavior to the full model and fits well.

**Fig. I. No-boundary avoidance (“no BA”) model behavior.** A variant of the full model without warped tuning curves (i.e., without efficient encoding / boundary avoidance) reproduces human behavior in the discrimination task, but has much smaller biases in the estimation task (panels B and D). As a result, the relationship between signed estimation accuracy and discrimination accuracy is also different in the model than in the data, with higher discrimination accuracy than signed estimation accuracy. Inset, zoomed-in view shows that estimates are biased, but only by a few degrees. **Fig. J. No-conditional-inference (“no CI”) model behavior.** A variant of the full model without conditional inference captures human discrimination accuracy well, and captures estimation means well, but fails to capture the bimodal shape of the estimate distribution (panel B).

**Fig. K. Model comparison.** Models are modified versions of the full model, with: no gain change; no boundary repulsion; no conditional inference; replacement of gain changes with tuning changes (TC); and replacement of gain changes with tuning changes but without boundary repulsion or conditional inference (TC_reduced_) [11,12]. **A.** Goodness-of-fit of each model (i.e., training set / in-sample loss), averaged across iterations of model fitting (102) and observers (N = 14). Error-bars, 2 SEM across observers. **B.** Generalization performance of each model (i.e., test set / out-of-sample loss), averaged across iterations of model fitting (102) and observers (N = 14). Error-bars, 2 SEM across observers. Ultimately, we selected the full model (baseline in this figure) as the winning model.

**Fig. L. Example boundary avoidance templates, for different values of *w_b_* and *σ_b_***. Intuitively, these curves represent the extent to which the observer “avoided” the horizontal (0º) boundary in their estimation responses, and are formally defined as the derivative of the warping function that is used to re-map the sensory neurons’ tuning curves.

**Fig. M. Efficient coding / tuning changes model, “TC_reduced”**. A model variant in which we assumed training-induced tuning changes like in the TC model, but without boundary avoidance, gain change, and conditional inference. This model variant fails to capture the magnitude of the estimation biases and their change with learning in the data.
